# BCR–ABL1 Drives Transcriptional Reprogramming of Chronic Myeloid Leukemia Cells for Immune Evasion Through C/EBPβ

**DOI:** 10.1002/mco2.70747

**Published:** 2026-04-26

**Authors:** Xiaocui Lu, Hui Fang, Yuan Liu, Chang Liu, Xuexiu Fang, Atsuko Matsunaga, Stephanie F. Mori, Ting Zhang, Gavin Wang, George I. Zhou, Miao Yu, Haocheng Ding, Jorge Cortes, Bo Cheng, Tianxiang Hu

**Affiliations:** ^1^ Department of Stomatology Zhongnan Hospital of Wuhan University Wuhan China; ^2^ Georgia Cancer Center Augusta Georgia USA; ^3^ Department of Dermatology Tianjin Academy of Traditional Chinese Medicine Affiliated Hospital Tianjin China; ^4^ Department of Biostatistics, Data Science and Epidemiology, School of Public Health Augusta University Augusta Georgia USA; ^5^ Immunology Center of Georgia Augusta Georgia USA; ^6^ Department of Biochemistry and Molecular Biology Medical College of Georgia Augusta Georgia USA

**Keywords:** BCR–ABL1, chronic myeloid leukemia, neutrophils, immune evasion, tumor microenvironment

## Abstract

Emerging immunotherapy holds promise to achieve treatment‐free remission (TFR) for chronic myeloid leukemia (CML) patients, the development of which depends on full understanding of mechanisms driving immune evasion. Our current investigation in a mouse CML model revealed dominant presence of neutrophils during CML progression, accompanied by significant reductions and exhaustion of T cells. In coculture, these BCR–ABL1 expressing neutrophil‐like CML cells significantly inhibited T cell proliferation. Gene expression profiling revealed that there was a global activation of both neutrophil markers and related immune suppression genes in these CML cells. Correlative analysis revealed strong correlations between the expression of BCR–ABL1 and immune suppression genes, suggesting a potential regulation of those genes by BCR–ABL1. Importantly, we identified CEBPB as a critical transcription factor that directly regulated the expression of master immune modulators TGFB1 and ARG2 through promoter binding, in both human and mouse CML samples. Therefore, blocking BCR–ABL1, or its downstream C/EBPβ, TGF‐β and arginase with inhibitors or shRNAs rescued T cell suppression by neutrophil‐like CML cells. Accordingly, combination treatment with targeted therapy using ponatinib and immunotherapy with anti‐PD1 antibody not only provides rapid remission, but also delayed relapses after treatment discontinuation, justifying combination treatment for TFR of CML.

## Graphical abstract

There is global immunosuppression during CML initiation and progression, which is directly driven by BCR–ABL1 expressing CML cells. On one hand, the BCR–ABL1 oncogene drives the differentiation of leukemia cells toward the neutrophil lineage. On the other hand, the oncogene also transcriptionally activates master immune regulators, including arginase and TGF‐β, through C/EBPβ. Therefore, combination therapy targeting both leukemia cells and exhausted T cells provides rapid remission and delayed relapse in preclinical mouse CML model

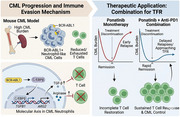

## 1 Introduction

Several lines of evidence suggest a pivotal role for the immune system in preventing and eliminating various types of tumors. Despite immune surveillance, tumors still manage to develop through genetic evolution facilitating immune resistance or immune suppression, a process known as immune evasion [1, 2, 3, 4]. Although some progress has been made in solid tumors, less is known about the mechanisms of immune evasion in hematological malignancies. As curative treatments for blood cancers, allogeneic hematopoietic stem cell transplantation (allo‐HCT) and donor lymphocyte infusions take advantage of the graft‐antileukemia effect to eliminate cancer cells [5, 6, 7, 8, 9]. This confirmed the dysfunction of the immune system in hematological malignancy and demonstrates that restoring antitumor immunity can maximize the benefits of treatment. In solid tumor, discovery and characterization of the critical function of PD‐L1/PD1 and CTLA‐4 in immune evasion lead to the development of checkpoint blockade therapies and provide cure for some cancer patients [10, 11, 12, 13, 14, 15, 16, 17, 18]. Therefore, a better understanding of the mechanisms underlying immune evasion in leukemia holds the promise to develop better treatment for patients with hematological malignancy and improve of their treatment outcome [19].

As the hallmark of chronic myeloid leukemia (CML), the *BCR–ABL1* fusion gene is the driver of the disease in CML patients [20, 21, 22]. The discovery of imatinib (Gleevec/Glivec) in the late 1990s for specifically targeting BCR–ABL1 kinase is a milestone of targeted therapy for cancer in human history [23, 24, 25]. Currently, oral tyrosine kinase inhibitors (TKIs) targeting BCR–ABL1 are widely used as the first‐line treatment for CML and can help achieve long‐term disease control in the majority of patients with CML [26, 27]. However, there are still challenges in CML patient management, including progression into accelerated or blast phase in a small but significant percentage of patients, emergence of drug resistance, and relapse after elective treatment discontinuation [21, 26, 28, 29]. For example, up to 20–30% of patients enrolled in frontline clinical trials discontinue therapy because of resistance [30]. The 5‐year survival rate for patients diagnosed in the accelerated phase or blast phase is still in the range of 30–50%. Noticeably, approximately 50% of CML patients who achieved deep molecular response after TKI treatment will relapse after TKI discontinuation [30, 31]. There is still an unmet need, therefore, for the development of novel treatment for CML to achieve the ultimate goal of treatment‐free remission (TFR) and cure for all patients [32, 33].

Mechanisms of leukemia immune evasion are most well studied in relapses after allo‐HCT, leading to discovery of mechanisms including genomic loss or transcriptional downregulation of HLA molecules, activation of immune checkpoint ligands, enforcement of immunosuppressive enzymes, increased anti‐inflammatory cytokines, and decreased proinflammatory cytokines or growth factors [34, 35, 36, 37]. Recent research implies that the CML cells can actively escape immune surveillance through downregulating the expression of major histocompatibility complex (MHC)‐II and its master regulator Class II transactivator [38], or by establishing an immunosuppressive tumor microenvironment (TME) in CML, including (1) restricted differentiation of conventional dendritic cells, (2) increased monocytes and basophils expressing high levels of PD‐L1 [39], and (3) the expansion of an immunosuppressive cell population, including myeloid‐derived suppressor cells (MDSCs) and regulatory T cells [40, 41, 42]. Importantly, this immunosuppressive environment also affects treatment response, and those with deeper molecular response to TKI have restored immune effectors and decreased immune suppressors [43, 44, 45, 46]. A comprehensive investigation of the mechanisms of immunosuppression in CML, therefore, is not only important to improve the outcomes of patients with advanced or refractory disease, but also critical for achievement of TFR.

Here, we performed continuous monitoring of immune composition during CML induction in both a primary transgenic mouse model and a serial transplantation model, which revealed global suppression of all immune effector cells. We further show that CML cells adopt a neutrophil phenotype, with systemic activation of immune suppression genes, which are directly responsible for the suppression of T cells. In addition, we demonstrate that the *BCR–ABL1* oncoprotein directly orchestrates the expression of these immune suppressors through CCAAT/enhancer‐binding protein (C/EBPβ), which are confirmed in both human and mouse CML cells. Importantly, our results show that combination of targeted therapy targeting BCR–ABL1 and immunotherapy targeting PD‐1 can provide faster remission and delayed relapse.

## 2 Results

### 2.1 CML Progression in Mouse Models Is Accompanied by Global Immunosuppression

**FIGURE 1:**
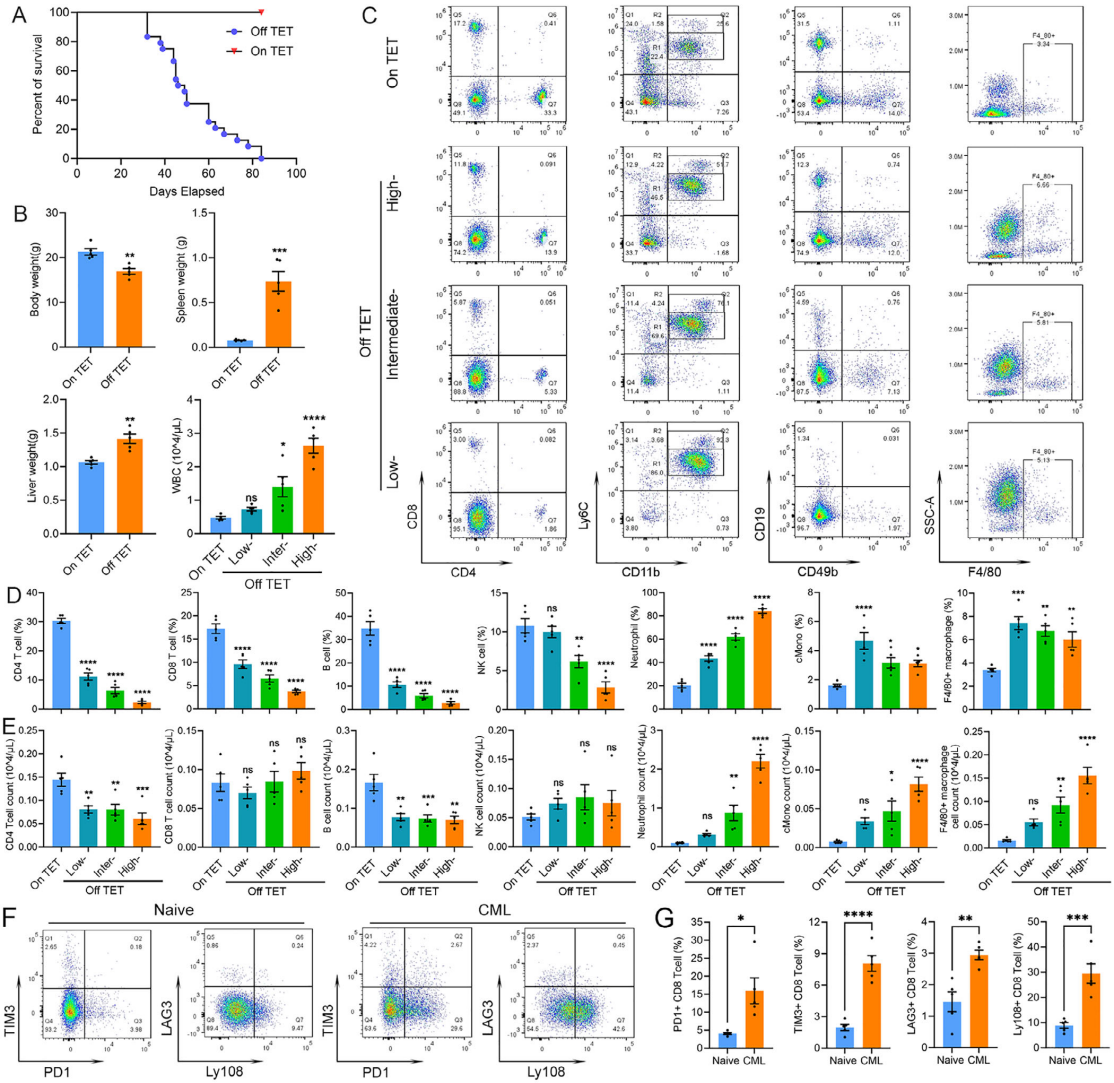
CML induction in *BCR–ABL1* transgenic mice is accompanied by a global immune suppression. Survival of the *BCR–ABL1* transgenic mice (*n* = 24) under continuous administration of tetracycline (On TET) or withdrawal of tetracycline (Off TET) from drinking water (A). Based on the percentages of the predominant neutrophils, CML progression in each mouse was classified into On TET (or naïve, <25%), low‐ (20–50%), intermediate‐ (50–75%), and late‐group (>75%) CML, as a reflection of leukemia burden. The body, spleen, and liver weights of mice at time of sacrifice and white blood cell count (WBC) at indicated disease stages (B). Representative plots of flow cytometry analysis of peripheral blood (PB) samples at indicated stages (mean ± SEM; *n* = 5). The corresponding statistical data for percentages and counts of indicated cell population are shown in panels (D) and (E). Representative dot plots for PD1, TIM3, LAG3, and Ly108 expression on CD8 T cells (F) and bar graphs of the calculated percentages (G) from naïve and CML mice. Data are presented as mean ± SEM (*n* = 5 biological replicates per group). The Student's *t*‐test was performed for comparison between two groups, whereas ANOVA was used for comparison of three or more groups. ns represents not significant; *p < 0.05, **p < 0.01, ***p < 0.001, ****p < 0.0001.

Withdrawal of tetracycline (TET) from the drinking water (Off TET) of the *SCL:tTA*/*BCR–ABL1* transgenic mice led to rapid CML progression after 32–84 days, with a median survival of 47.5 days (Figure 1A) as described previously [47]. In contrast, none of the control transgenic mice with continuous TET (On TET) administration showed any disease. The leukemia progression was evident by a continuous increase in white blood cell counts (WBC), significantly increased spleen and liver weights but decreased body weights at time of sacrifice (Figure 1B). Flow cytometry analysis of peripheral blood (PB) samples revealed that there was a predominant increase of neutrophils during CML progression [48, 49, 50], with an increase from ∼20.22 to 85.82% (Figure 1C,D). Therefore, we classified CML progression in mouse model into naïve, low‐, intermediate‐, and high‐disease burden with the cutoffs of <25%, 25–50, 50–75, and >75% (advanced disease with high mortality). The presence of classical monocytes and F4/80+ macrophages also increased when there was high CML burden (Figure 1C–E) but was far less than the dominant neutrophils. Immune effector cells, including CD4 and CD8 T cells, B cells, and NK cells, were all significantly reduced both in percentages and in absolute numbers, with the only exception of the NK cell absolute count (Figure 1C–E).

There is a potential argument that after TET withdrawal, the hematopoietic stem cells (HSCs) initiate BCR–ABL1 fusion oncoprotein expression and adopt a leukemic differentiation path instead of normal hematopoiesis, which leads to the reduced production of immune effector cells from HSCs in the primary CML induction model. Therefore, some of the observed “immunosuppression effects” in the above primary transgenic model could arise from leukemic transformation of HSCs or other transgenic mouse specific effects. To exclude this possibility, we performed serial transplantation of the primary CML cells into wild‐type C57BL/6 recipient mice with normal hematopoiesis and intact immune system (Figure S1A), which provides a clean CML model to test whether the BCR–ABL1 expressing CML cells can actively suppress immune surveillance to ensure leukemia progression. While advanced CML developed after 53–95 days in the recipient mice, there is a reduction in body weight and an increase in WBC, spleen, and liver weight (Figure S1B,C), which confirmed successful CML progression. At the time of sacrifice, flow cytometry analysis of the PB leukocytes showed a significant increase of the neutrophils in both the percentages and cell counts (Figure S1D–F). Meanwhile, almost all the immune effector cells, including CD4 and CD8 T cells, B and NK cells, displayed significant reduction in the percentages, compared with the control mice. CD4 T cell and B cell also show highly significant reduction in numbers in the leukemic mice (Figure S1E), confirming the same discovery in the primary CML induction.

Besides a significant reduction of CD4 and CD8 T cells in leukemic mice, flow cytometry shows that the remaining T cells express the T cell exhaustion marker PD1, TIM3, and LAG3 [51, 52, 53]. The PD1+, TIM3+, and LAG3+ CD8 T cells increase from 4.08, 1.97, and 1.46% in naïve mice to 15.91, 8.07, and 2.95% in leukemic mice, respectively (Figure 1F,G). In addition, intracellular staining for immune stimulatory cytokines revealed that those PD1+ CD8 T cells showed significantly reduced capabilities in producing IFNγ and TNFα (Figure S1G), further confirming the PD‐1^+^ cells are truly functionally impaired. Therefore, our results demonstrated that in both the primary induction and secondary transplantation CML models, CML cells actively establish an immunosuppressive TME, with predominant induction of neutrophils, and suppression of all immune effector cells, to ensure CML progression. In consistency, it has been reported that there is a significantly expanded granulocyte or neutrophil population in PB of CML patients at diagnosis [40, 41, 42]. In addition, PD‐1 expression levels are upregulated on CD8 T cells from CML patients and become significantly lower in those with deep molecular responses to TKI [43, 44, 54]. Therefore, the immune evasion phenomenon observed here highly recapitulates the process happening in CML patients.

### 2.2 The Dominant Neutrophils Represent BCR–ABL1 Expressing CML Cells

**FIGURE 2:**
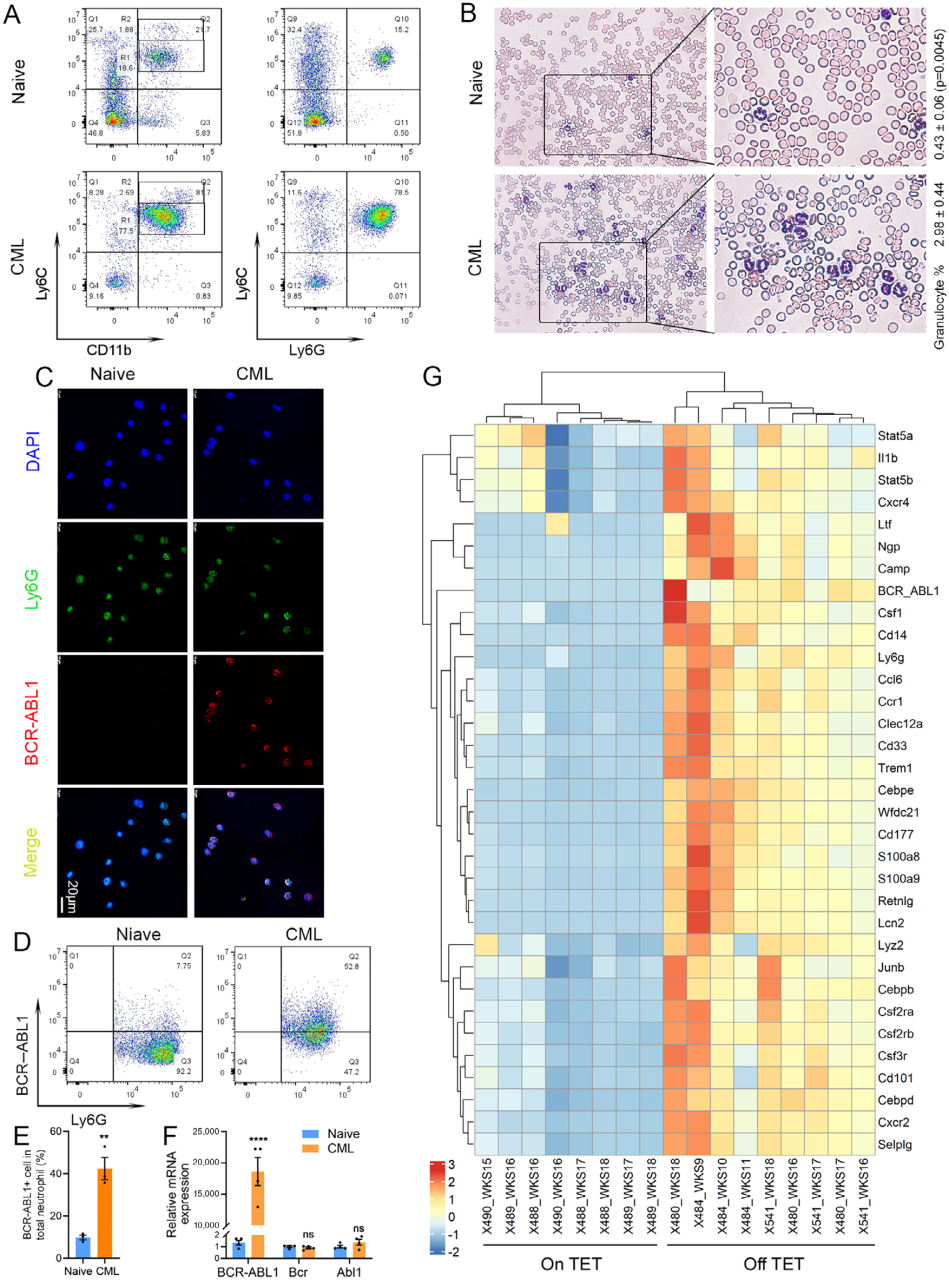
The predominant neutrophils represent *BCR–ABL1* expressing CML cells. Representative dot plots of flow cytometry show the dominant CD11+Ly6C^Int^Ly6G+ neutrophils in CML mice (A). May‐Grunwald Giemsa Staining of blood smears revealed a granulocyte morphology of the majority of leukocytes in the PB of CML mice, and the percentages of granulocytes in different conditions were provided on the right side of the images (B). Images from immunofluorescence (IF) staining of purified neutrophils from naïve and CML with DAPI and antibodies against Ly6G and BCR–ABL1 oncoprotein (C). Flow cytometry data from intracellular staining of BCR–ABL1 in the gated Ly6G+ neutrophils from naïve and CML mice (D), and the percentages are shown in panel (E; *n* = 4). qRT‐PCR detection of the transcription levels of the human *BCR–ABL1* transgene and endogenous mouse *Bcr* and *Abl1* genes in the purified neutrophil samples with three biological replicates (F). Heatmap plot of the expression of the listed neutrophil marker genes in PB samples from control (On TET) and leukemic (Off TET) mice at indicated weeks (G). X488, X489, and X490 are three control mice on continuous tetracycline administration. X480, X484, and X541 are three leukemia mice after withdraw of tetracycline. WKS represents weeks. RNA‐Seq data from Weeks 16–18 are used for the analysis, with the exception of X484 using samples from Weeks 9–11, due to rapid CML progression.

The progression of leukemia is usually accompanied by the presence of large numbers of leukemic blast cells in the peripheral circulation. According to our flow cytometry data, neutrophils dominate the circulatory leukocytes in late stage CML, with a percentage of 85.82% compared with 20.22% in the naïve mice (Figures 1C–E and 2A). This observation is confirmed by Giemsa staining of blood smears, with a significantly increased presence of polymorphonuclear granulocytes or neutrophils, from 0.43% in naïve mice to 2.98% in CML cohort (Figure 2B). The predominant presence of CD11b+Ly6C^Int^Ly6G+ neutrophil in the PB, bone marrow (BM), and spleen implies that these cells may overlap with CML cells. Indeed, immunofluorescence (IF) staining with these purified neutrophils revealed that the BCR–ABL1 protein only showed a colocalization with Ly6G in the CML samples, but not in the naïve control mice (Figure 2C). Flow cytometry with intracellular staining for the BCR–ABL1 fusion protein confirmed 42.2% of neutrophils in the CML mice expressed the oncoprotein (Figure 2D,E). qRT‐PCR analysis using primers against the *BCR–ABL1* fusion transcripts also revealed that the expression of *BCR–ABL1* mRNA was 18,619.3 times higher in neutrophils from the leukemic mice compared with the naïve counterparts, confirming the presence of CML hallmark BCR–ABL1 in the neutrophils (Figure 2F). In contrast, there were no significant differences in the expression levels of endogenous mouse *Bcr* or *Abl* genes between the naïve and CML cohorts. In addition, we also investigated the expression of neutrophils markers in RNA‐Seq data of PB samples with advanced CML between 11 and 18 weeks or the matched controls [55]. Mice with identification number X488 (Weeks 16–18), X489 (Weeks 16–18), and X490 (Weeks 15 and 16) represent the On TET control group, and mice X480 (Weeks 16–18), X484 (Weeks 9–11), and X541 (Weeks 16–18) are from TET‐off (Off TET) CML cohort. As shown in the heatmap, the mRNA expression of the *BCR–ABL1* fusion oncogene is significantly increased as expected in the Off TET leukemic cohort (Figure 2G). Meanwhile, the marker genes *Ly6g*, *Cd101*, *Cd177*, *Clec12a*, *Cd33*, *Ltf*, *Lcn2*, *Ngp*, *Camp*, *Selplg*, *Retnlg*, *Ccr1*, *Cxcr2*, *Cxcr4*, *Il1b*, *Lyz2*, *S100a8*, and *S100a9*, and regulator genes in neutrophil differentiation, including *Junb*, *Cebpb*, *Cebpd*, *Csf1*, *Csf2ra*, *Csf2rb*, *Csf3r*, and *Trem1* [56, 57, 58, 59, 60, 61, 62, 63], are all upregulated, indicating a transcriptional reprogramming toward neutrophil differentiation in BCR–ABL1+ CML cells. Therefore, we confirm that the *BCR–ABL1* expressing CML cells present the same gene signature and immunophenotype as the neutrophils in the mouse CML models. This phenomenon is also supported by the granulocytic predominance and expression of markers for myeloid lineage cells, particularly granulocytes and their precursors in CML patients, including CD10, CD11b, CD13, CD15, CD16, CD33, and MPO [40, 41, 42, 64, 65, 66, 67].

### 2.3 The Neutrophil‐Like CML Cells Perform an Immunosuppression Function

**FIGURE 3:**
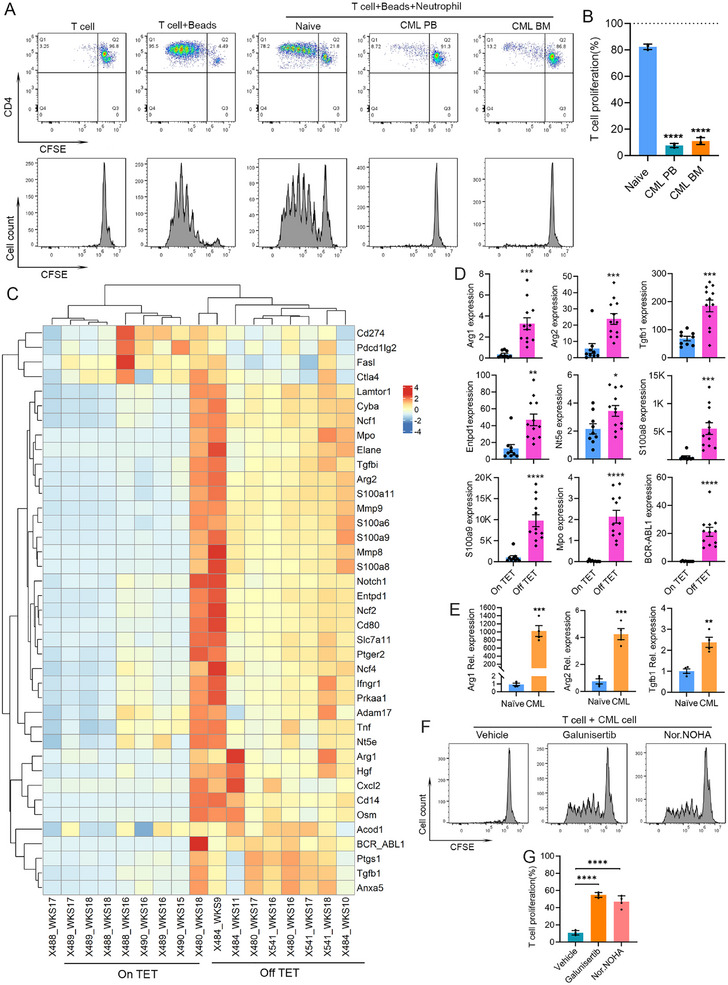
The neutrophil‐like CML cells perform immune suppression function with activation of immune suppression genes. Flow cytometry data of CD4 T cells cultured under different conditions: T cell only, with bead stimulation, with bead stimulation plus indicated neutrophils from PB and BM of CML mice or BM of naïve mice at a 1:1 ratio (A). Data are calculated from three independent experiments. The cell division in T cell with bead stimulation was considered as 100% and the relative proliferation activities for the others were calculated (B; *n* = 3). Heatmap plot of the expression of the listed immune suppression genes in PB samples from control (On TET) and leukemic (Off TET) mice at indicated weeks (C). The expression levels of the selected genes in Fragments Per Kilobase of transcript per Million mapped reads (FPKM) were plotted as bar graphs (D; *n* = 9 for naïve and 12 for CML groups, respectively). (E) qRT‐PCR detection of the relative expression of the indicated genes with three biological replicates. T cell proliferation during coculture with neutrophil from CML mice pretreated with vehicle, 10 µM TGF‐β inhibitor galunisertib or 30 µM arginase inhibitor nor‐NOHA acetate or vehicle control, with representative histogram plots in panel (F) and statistical data in panel (G; *n* = 3).

The next question we asked was whether these neutrophil‐like CML cells performed any immunosuppression functions. The purified CML cells were cocultured with CD4 T cells in the presence of anti‐CD3/CD28 beads. When cocultured at a 1:1 ratio, 82.33% of the T cells incubated with neutrophils from naïve mice divided, while only 7.67 and 11.00% T cells cocultured with neutrophils from the leukemic mice PB and BM, respectively, showed cell division, indicating a strong potency of these neutrophil‐like CML cells in suppressing T cell proliferation (Figure 3A,B).

To investigate the mechanisms underlying neutrophil‐like CML cell‐mediated immunosuppression, we also investigated the expression of immunosuppression‐related genes in RNA‐Seq data of *BCR–ABL1* CML samples [55]. As shown in the heatmap and bar graph (Figures 3C,D and S2A), the mRNA expression of the most neutrophil‐related immunosuppressive pathway genes, such as *Arg1*, *Arg2*, *Tgfb1*, *Enptd1*, *Nt5e*, *S100a8*, *S100a9*, *Ptgs1*, *Mmp8*, and *Mmp9*, are increased when the *BCR–ABL1* fusion oncogene is activated in the Off TET leukemic cohort. It is noticeable that several well‐known immune checkpoint molecules, including *Cd274*, *Ctla4*, and *Pdcd1lg2*, do not show any clear differences between the two cohorts. The activation of immunosuppressive genes *Arg1*, *Arg2*, and *Tgfb1* was further confirmed by qRT‐PCR using neutrophils purified from leukemic and control naive mice (Figure 3E). It is noticeable that although *Arg1* shows more folds of increased expression compared with *Arg2*, the latter has higher absolute expression level, as revealed by RNA‐Seq. These data confirmed that in the neutrophil‐like CML cells, the immunosuppressive genes were significantly activated, which might contribute to immunosuppression.

Based on the absolute expression levels revealed by RNA‐Seq and their critical roles in immunosuppression, we selected the arginase and TGF‐β pathways for further validation of function on T cell suppression. Galunisertib (LY2157299), a selective inhibitor for TGF‐β receptor Type I (TGF‐βRI) kinase [68, 69], and nor‐NOHA acetate [70, 71], a selective and reversible arginase inhibitor, were used for pretreatment of neutrophil‐like CML cells and then T cell coculture was performed. The results showed that blocking either pathway can partially restore T cell proliferation, with galunisertib displaying relative higher efficacy (Figure 3F,G). These results support that the arginase and TGF‐β pathways are at least partially responsible for the immune suppression mediated by the BCR–ABL1+ CML cells during leukemia progression.

### 2.4 BCR–ABL1 Oncoprotein Actively Regulates the Expression of Immunosuppression Genes

**FIGURE 4:**
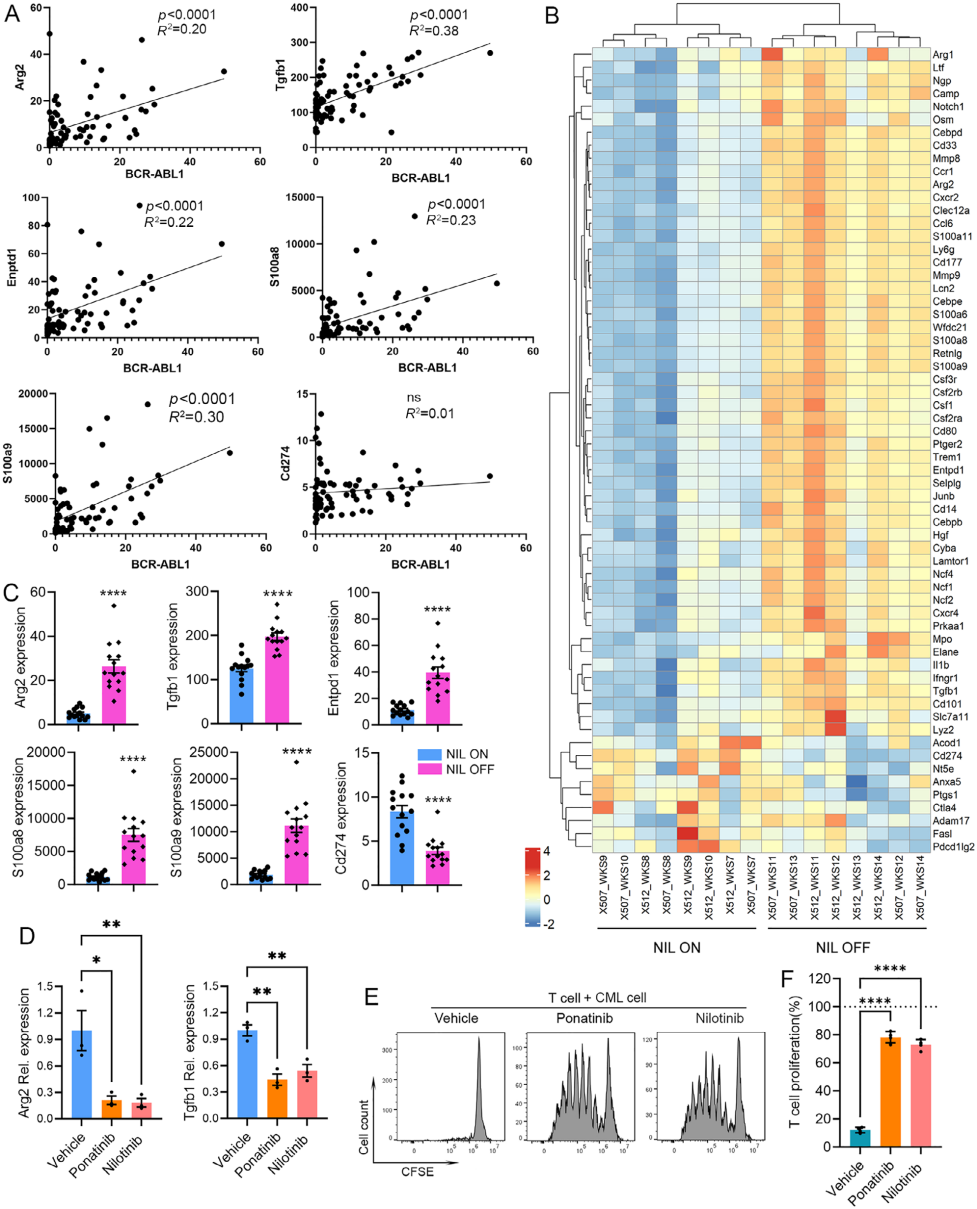
BCR–ABL1 oncoprotein actively regulates the expression of immune suppression genes. (A) The correlations between the mRNA expression levels of *BCR–ABL1* and selected immune suppression genes were analyzed using the gene expression profiling data of transgenic mice through CML induction from Week 0 to 18 or until mice became moribund with leukemia after TET withdrawal. (B) Heatmap plot of the expression of the listed neutrophil markers and immune suppression genes in PB samples from leukemic mice under the 4 week nilotinib treatment window (NIL ON) and another 4 weeks after discontinuation (NIL OFF). The expression levels of the selected genes in FPKM were plotted in bar graphs (C; *n* = 14). qRT‐PCR detection of indicated genes in purified neutrophil from CML mice with and without ponatinib or nilotinib treatment (D; *n* = 3). T cell suppression by neutrophil from CML mice pretreated with vehicle, 30 nM TKI ponatinib or 150 nM nilotinib, with (E) showing the flow cytometry plots and (F) for statistical data of T cell proliferation activities. Data are calculated from four independent experiments.

The heatmap plot using the RNA expression data for the PB samples of advanced CML suggests a potential regulation of neutrophil differentiation and immune suppression genes by *BCR–ABL1*. Therefore, we first performed a correlation analysis between the expression levels of *BCR–ABL1* and immunosuppressive genes using the RNA‐Seq data covering the whole process of CML induction, from Week 0 with TET to Week 18 after TET withdraw, where the *BCR–ABL1* gene showed gradual activation [55]. As shown in Figures 4A and S2B, highly significant positive correlations were detected between the expression levels of *BCR–ABL1* and immunosuppression genes *Arg1*, *Arg2*, *Tgfb1*, *Enptd1*, *Elane*, *S100a8*, *S100a9*, *Mpo*, *Ptgs1*, *Mmp8*, and *Mmp9*, but not *Cd274*, indicating a potential direct regulation of these target genes by *BCR–ABL1*. We further investigated whether blocking BCR–ABL1 activity with TKI nilotinib can impair the activation of these immunosuppression genes. RNA‐Seq data of murine samples from the 4‐week treatment window (NIL ON, Week 7–10) and 4 weeks after discontinuation (NIL OFF, Week 11–14) were used for both heatmap and box plots (Figures 4B,C and S2C). Most examined genes including markers for neutrophil, such as *Ly6g*, *Cd101*, *Cd177*, *Ngp*, *Camp*, *Lcn2*, and *Csf3r*, as well as genes involved in neutrophil‐mediated immune suppression, such as *Arg1*, *Arg2*, *Tgfb1*, *Enptd1*, *Elan*, *S100a8*, *S100a9*, *Mpo*, *Cd14*, *Mmp8*, and *Mmp9* showed increased expression levels after withdrawal of nilotinib, indicating a direct regulation of these genes by oncoprotein BCR–ABL1. However, *Cd274*, *Pdcdlg2*, *Nt5e*, and *Ptgs1* displayed a decreased expression after BCR–ABL1 activity is restored, suggesting an indirect regulation of these targets by oncoprotein.

We then used nilotinib and ponatinib to block the activity of BCR–ABL1 in the purified neutrophil‐like CML cells, and the mRNA expression of immunosuppressive genes was detected by qRT‐PCR. The results showed that after blocking BCR–ABL1 activity, the expression levels of *Arg1*, *Arg2*, and *Tgfb1* were significantly decreased (Figure 4D). Most importantly, when CML cells were pretreated with either nilotinib or ponatinib, their potency in T cell suppression was dramatically reduced, with significantly restored T cell proliferation (Figure 4E,F). Therefore, these results confirm a direct role for BCR–ABL1 signaling in activating immunosuppressive genes to suppress immune effector cells.

Our results from mouse CML models revealed a novel role of BCR–ABL1 in actively modulating the TME through upregulation of immune suppression genes during leukemogenesis. We then examined whether the same regulation exists in human CML cells. Using the public ENCODE data at Genome Browser, we show that the critical target genes *TGFB1* and *ARG2* are actively expressed in widely used CML cell line K562, with *TGFB1* at a relatively higher level (Figure S3A). In consistent, chromatin immunoprecipitation (ChIP)‐Seq data show both *TGFB1* and *ARG2* promote are associated with active histone modifications H3K4m3 and H3K27ac, and the gene promoters and bodies are also occupied by RNA polymerase II, confirming that this BCR–ABL1‐mediated active immune modulation is conserved in both mouse and human models.

### 2.5 BCR–ABL1 Coordinates the Expression of Immunosuppression Genes Through C/EBPβ

**FIGURE 5:**
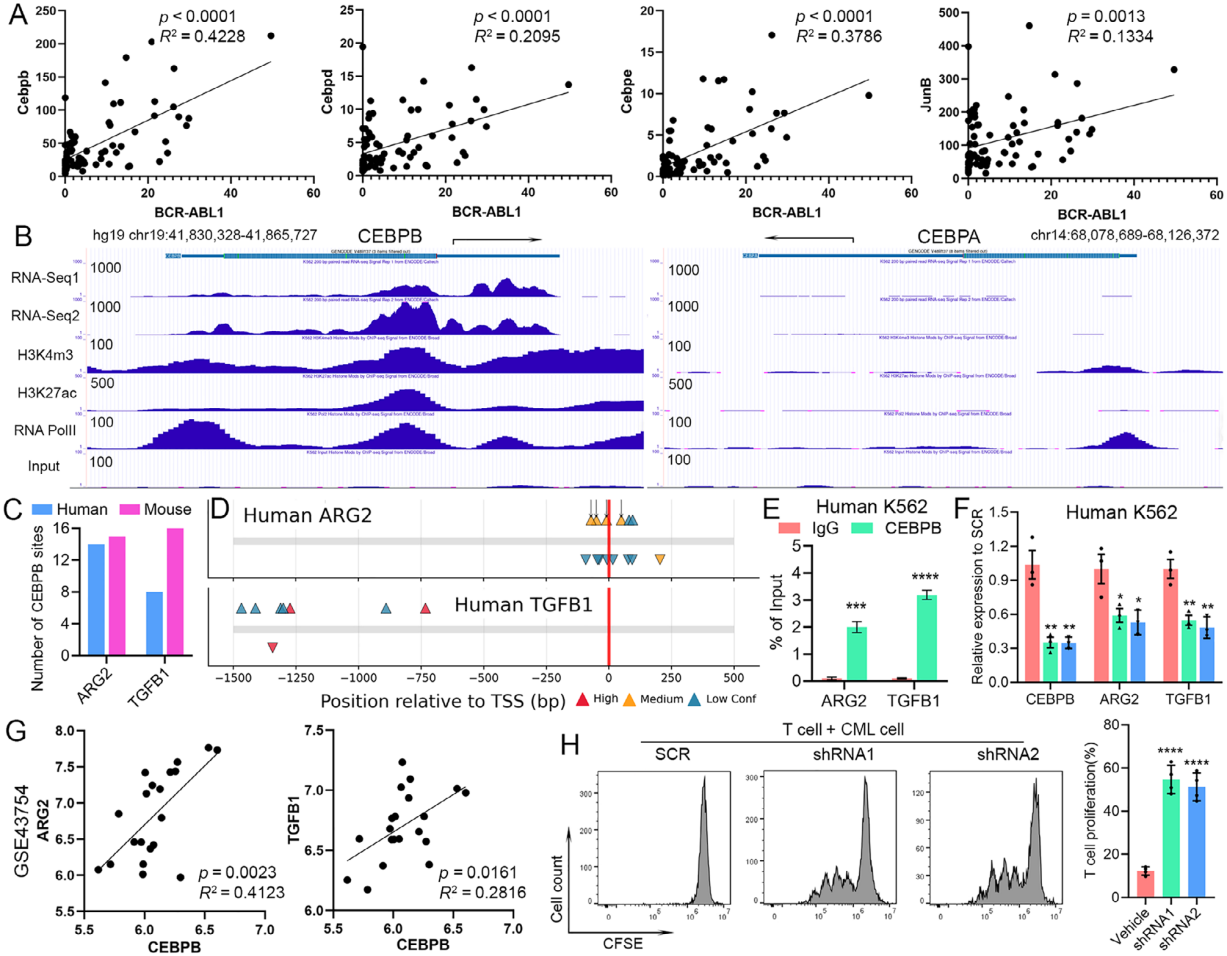
BCR–ABL1 regulates the expression of immune suppression genes through C/EBPβ. (A) Correlations between the mRNA expression levels of *BCR–ABL1* and selected critical neutrophil transcription factors. (B) Genome browser snapshot of the *TGFB1* locus showing tracks of RNA‐Seq data, and ChIP‐Seq data with histone markers H3K4m3 and H3K27ac, and RNA PolII from K562 cells. (C) Bar graph showing the numbers of potential CEBPB binding sites in the indicated promoters of human and mouse genes. (D) Diagram showing the locations of the predicted CEBPB binding sites in the promoter regions of human ARG2 and TGFB1 genes. (E) ChIP‐qPCR quantification of the enrichment of target genomic DNA relative to the total input DNA after pulling down using CEBPB antibody or IgG control. (F) qRT‐PCR detection of *CEBPB*, *ARG2* and *TGFB1* in K562 after knockdown of *CEBPB* using two different shRNAs, with the scramble (SCR) construct transduced cells as the control. Three biological replicates were included in each group in panels (E) and (F). Correlations between the mRNA expression levels of CEBPB and ARG2 or TGFB1 in a CML patient cohort with GEO accession number GSE43754 (G). T cell suppression by primary mouse CML cells with or without knockdown of *Cebpb*. The representative flow cytometry plots and statistical data of T cell proliferation are shown in panel (H; *n* = 4). TSS, transcription starting site. Red, yellow, and blue triangles represent those predicted binding motifs with high, medium, and low confidence, respectively.

Our next question is how BCR–ABL1 oncoprotein coordinates the activation of multiple immune regulator genes in neutrophil‐like CML cells. It has been reported that key transcription factors (TFs), including JUNB in the AP‐1 complex and C/EBP family members, are implicated in emergency granulopoiesis and inflammatory response in neutrophils [59, 72, 73, 74, 75]. In consistency, *Junb*, *Cebpb*, *Cebpd*, and *Cebpe* all show activation upon CML induction (Figure 2G), and their expression levels are reduced when BCR–ABL1 signal is blocked by nilotinib (Figure 4B), showing potential expression regulation by BCR–ABL1. Indeed, correlation analysis revealed that there are significant correlations between the expression levels of *BCR–ABL1* and *Junb*, *Cebpb*, *Cebpd*, or *Cebpe* (Figure 5A), with *Cebpb* showing the strongest correlation with R‐squared value of 0.4228 and as the most highly expressed C/EBP family member. However, only *JUNB* and *CEBPB* are actively expressed and associated with active chromatin markers in human CML cell line K562 (Figures 5B and S3B). In comparison, *CEBPA*, which is reported to be suppressed by BCR–ABL1 in CML cells [76, 77, 78], is not associated with active markers and not expressed. Considering the role of C/EBPβ in regulating both TGF‐β and arginase [79, 80], we focus on testing the hypothesis that whether BCR–ABL1 orchestrates a transcriptional reprogramming for immune suppression through C/EBPβ.

Analysis of the promoter sequences of both human and mouse *ARG2* and *TGFB1* genes revealed the high abundances of CEBPB binding motifs (Figure 5C), and the distribution of those sites were shown in their perspective promoter maps (Figures 5D and S3C). To confirm this direct promoter occupancy, ChIP‐qPCR was performed and there was significantly increased pull‐down of DNA fragments with condensed CEBPB binding motifs when using the CEBPB antibody compared with the IgG control (Figures 5E and S3D). We then used short‐hairpin RNA (shRNA) to knockdown *CEBPB* in both human K562 and mouse primary CML cells and examined the expression of *ARG2* and *TGFB1*. In human CML cells, the two different shRNAs show similar efficiencies in knocking down target gene *CEBPB*, and the mRNA expression levels of both *ARG2* and *TGFB1* are significantly reduced (Figure 5F). In mouse primary CML cells from our transgenic mouse CML model, *Cebpb* knockdown showed similar effects on the expression of *Arg2* and *Tgfb1* as in the human CML cells (Figure S3E). In addition, significant correlations between the expression levels of CEBPB and ARG2 or TGFB1 are observed in a CML patient cohort (Figure 5G). Similar positive correlations are found in mouse CML samples (Figure S3F). In consistency, these neutrophil‐like CML cells from leukemic mice showed reduced potencies in suppression of T cell proliferation in coculture, when C/EBPβ expression was silenced by shRNAs (Figure 5H). Altogether, our data support a critical role of neutrophil TF CEBPB in activating immune suppression‐related genes *ARG2* and *TGFB1* in both human and mouse CML cells, and this BCR–ABL1/C/EBPβ/arginase and TGF‐β axis is important for immune evasion in CML.

### 2.6 Combinational Targeting BCR–ABL1 and PD1 Leads to Rapid Disease Control and Attenuated Relapses

**FIGURE 6:**
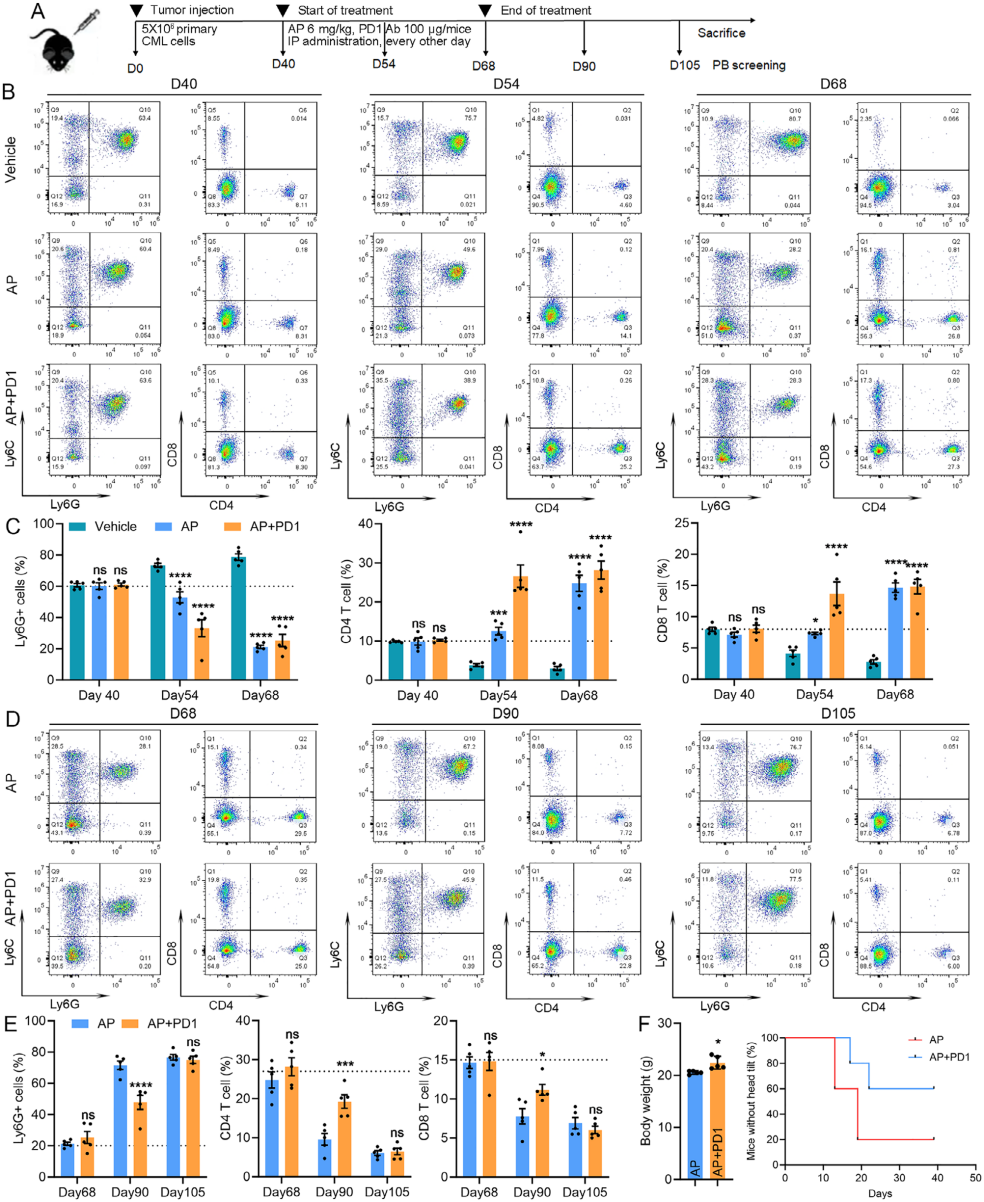
A combination treatment targeting CML cells and PD1 provides better CML outcome. Diagram of the experimental outline with drug treatment and PB screen schedules (A). Representative plots of flow cytometry analysis of PB from different mouse cohorts, including vehicle control, ponatinib treatment (AP), and AP in combination with anti‐PD1 antibody (AP+PD1) at indicated time points (B) and (D). The statistical results for percentages of indicated cell types are shown in panel (C; *n* = 5) and (E; *n* = 5). The body weights of mice at time of sacrifice and percentages of mice showing head tilt during the time course are shown in panel (F; *n* = 5).

Our above results suggest that the BCR–ABL1 oncoprotein actively drives an immunosuppression reprogramming inside the neutrophil‐like CML cells, which was accompanied by the presence of PD1+ exhausted T cells (Figure 1F–H). Then, we tested whether blocking T cell exhaustion can improve treatment outcome, and we compared the therapeutic efficacy of ponatinib treatment alone or in combination with an anti‐PD1 antibody using CML mouse model. The treatment regimen is shown in Figure 6A. Forty days after primary CML cell inoculation, there was a high percentage (∼60%) of neutrophil‐like CML cells in all leukemic mice. After 2 weeks of treatment, the CD4 and CD8 T cells significantly increased in the combinational therapy group compared with both the single treatment and vehicle controls (Figure 6B,C). Accordingly, this group showed the best disease control, with the lowest percentages of Ly6G+ CML neutrophils in the PB. These treatment benefits become almost undetectable after prolonged treatment for 4 weeks, supporting ponatinib itself as a highly competent targeted therapy.

It is reported that restored T cell function is related to better CML control and reduces the likelihood of relapse [43, 44, 81]. To determine whether this combinational treatment provides any benefits in preventing relapses, we discontinued all treatments on Day 68, when the ponatinib alone and ponatinib plus anti‐PD1 combination groups achieved almost the same complete remission (Figure 6D,E). At this moment, the mice in the vehicle group were sacrificed due to advanced leukemia, thus they were not included in the comparison. Three weeks after treatment discontinuation, the TKI only group displayed rapider leukemia relapses, with significantly higher Ly6G+ CML cells and lower percentages of CD4 and CD8 T cells compared with combinational treatment. These benefits in immunological control provided by combinational treatment became undetectable after 5 weeks of treatment discontinuation. It is noticeable that these CML mice show head tilt syndrome, which is related to high CML burden and a potential indication of invasion of CML cells into the central nervous system. In the combination treatment, 60% of the mice did not show any head tilt during the observation period, compared with only 20% of mice in the ponatinib only cohort, further confirming a better disease control and delayed relapse after discontinuing treatment (Figure 6F). Therefore, we demonstrate that a combination treatment of PD1 and TKI targeted therapy not only provides rapid remission, but also delayed relapses in CML model, warranting further investigation in combining TKI and immune checkpoint inhibitors for TFR of CML.

## 3 Discussion

The development of TKI has completely shifted the treatment paradigm of CML, with the remarkable achievement of overall 5‐year survival rate of over 70%. However, patients diagnosed in the accelerated phase or blast phase have lower 5‐year survival rate of 30–50% [26, 28, 29]. In addition, TFE, the modern primary goal for many patients, is a reality in only approximately 25–30% of all patients [45, 82, 83]. There is emerging evidence that the key to full remission is restoring a functional immune surveillance [44, 45, 46, 84]. Therefore, a comprehensive understanding of mechanisms of immune evasion in CML holds the key to further improving CML patient outcomes. However, these mechanisms have been underinvestigated in the era of TKI. Previous immune cell compositions in CML patients have demonstrated the increased presence of neutrophils or polymorphonuclear (PMN)–MDSCs and a potential role of mesenchymal stem cells in activating neutrophils in T cell suppression in the BM microenvironment [40, 41, 42, 43]. However, those studies usually are based on limited sampling from different patients with heterogeneous background and variable disease burdens, which can reveal some correlations but impairs in depth mechanistic studies. Taking advantage of the inducible transgenic mouse CML model, this study provides the first comprehensive profiling of the dynamic changes of TME during primary CML induction and secondary transplantation, both of which confirmed a global immune suppression during leukemia progression. Importantly, we not only revealed this global immunosuppression during CML progression, but also demonstrated the detailed mechanisms underlying the establishment of immunosuppression in CML. On one hand, the *BCR–ABL1* oncogene drives the differentiation of CML cells toward the neutrophil lineage. On the other hand, it also actively coordinates a transcriptional activation of multiple pathways involved neutrophil‐mediated immune suppression, including arginase, TGF‐β, adenosine, and S100a8/9. Correlation analysis of RNA‐Seq data, ChIP‐qPCR, and pharmacological or genetic inhibition in combination with T cell suppression assay demonstrated a novel role of the BCR–ABL1/C/EBPβ/arginase and TGF‐β axis in immune modulation in CML. Therefore, our research provides direct evidence that BCR–ABL1, the CML driver oncoprotein, actively reprograms the transcription activities inside CML cells to suppress the antitumor immunity and facilitates the immune evasion process.

It is noticeable that critical immune checkpoints, including *Cd274*, *Pdcd1lg*, and *Ctla4*, do not show specific activation upon CML induction, and there are also lack of correlations between their expressions and that of *BCR–ABL1*. Instead, multiple other immune suppressive pathways, including arginase, TGF‐β, adenosine, and prostaglandin E2, are activated in neutrophil‐like CML cells. Arginase‐1 was first demonstrated to be produced by MDSCs and negatively regulated T‐cell function in vivo [85, 86, 87]. Arginase‐2 shares the same activities as arginase‐1, with some differences in tissue preference and different subcellular localization [70, 88]. In addition, overexpression of *Arg2* has been reported as a compensatory mechanism for loss of *Arg1* [89]. Arginase plays crucial roles in modulating the antitumor immunity of T cells mainly through depletion of l‐arginine from extracellular space, leading to T‐cell dysfunction and impairment of anticancer immune responses. It has been reported that there are increased levels of arginase‐1 expressing MDSCs in CML patient samples [40, 90]. Here, we show that the predominant neutrophil‐like population in CML mice mainly express *Arg2* instead of *Arg1*, suggesting cell context‐dependent activation of different arginases.

TGFβ, primarily acting as an immunosuppressive factor, plays a critical role in suppression of antitumor immunity through multiple mechanisms [91, 92, 93]. In solid tumor, TGF‐β released by cancer‐associated fibroblasts play a critical role in T cell exclusion and inhibition of Th1 differentiation, which is important mechanism for resistance to immune blockade therapy [92, 94, 95]. However, the role of TGF‐β in hematological malignancies can be complicated, considering its roles in both immune modulation and differentiation regulation [96, 97]. Noticeably, some early studies in CLL, B‐ and T‐cell ALL, and HTLV‐I leukemogenesis suggested its role as a tumor suppressor, where leukemia cells found different ways to become resistant to TGFβ signaling [98, 99, 100, 101]. The immune modulation functions of TGFβ in myeloid leukemia is underappreciated and not well documented. It has been reported that BCR–ABL1 expressions significantly increase TGF‐β/Smad‐dependent transcriptional activity using in vitro cell lines [102]. Therefore, we not only identified arginase and TGF‐β as critical molecules produced by BCR–ABL1 CML cells in mediating immune evasion, but also demonstrated that BCR–ABL1 oncoprotein actively regulated their expression through key neutrophil TF C/EBPβ. Since it is always challenging to target a TF, our discovery of arginase and TGF‐β as direct downstream targets of C/EBPβ provides new options to reverse BCR–ABL1‐mediated immune evasion, by using small molecular inhibitors or antibodies for arginase and TGF‐β. In addition, direction blockage of T cell exhaustion mediated by the PD‐1 molecule can be another alternative to restore antitumor immunity in CML, as demonstrated in our preclinical mouse model. In this scenario, TKI‐resistant CML cells can be eradicated by restored antitumor immunity, preventing any relapse or recurrence driven by resistant leukemia cells. Therefore, a combination of targeted therapy and immunotherapy targeting those immune suppression pathways identified here will also help achieve the ultimate treatment goal for CML, a TFR.

BCR–ABL1 modulates the activity of TFs that regulate the expression of differentiation‐related genes during CML transformation. It has been reported that *BCR–ABL1* mRNA and protein levels are higher in blast phase compared with chronic phase of CML [21]. While low levels of BCR–ABL1 allow granulocytic maturation, increased BCR–ABL1 levels impair myeloid maturation of Philadelphia chromosome‐positive (Ph+) granulocyte‐macrophage progenitors [22]. One of the important mechanism is that high BCR–ABL1 levels lead to a remarkable downregulation of C/EBPα, a TF essential for granulocytic differentiation, through the stabilization of the poly(rC)‐binding protein heterogeneous nuclear ribonucleoprotein E2 (hnRNP E2) [21, 22, 76, 78]. Indeed, our data also show that C/EBPα was not barely expression in either mouse or human CML cells. However, our data revealed that C/EBPβ, another member of the C/EBP family, was highly active and expressed in both mouse and human models. While downregulation of C/EBPα impairs the terminal differentiation of myeloid progenitors into fully mature granulocytes or neutrophils, upregulation of C/EBPβ, a key mediator of the inflammatory response, is involved in the activation of immune suppression genes to inhibit antileukemia immunity of the host to ensure leukemia progression. Noticeably, induction of ectopic expression of either C/EBPα or C/EBPβ, but not C/EBPε, can drive granulocytic differentiation in K562 cells [103], supporting our discovery of C/EBPβ as a master regulator for BCR–ABL1‐driven neutrophilic transcriptional reprogramming. Cross referencing the phospho‐proteomic profiling of CML cells showed that C/EBPβ was not a direct target of BCR–ABL1 oncoprotein [104]. Therefore, it is unlikely that BCR–ABL1 kinase directly phosphorylates or stabilizes C/EBPβ to activate their downstream targets. Instead, it has been shown that BCR–ABL upregulated C/EBPβ indirectly through the activation of STAT5 [105].

In consistent with an immunosuppressive TME, the PD‐1+ exhausted T cells were detected in CML patients, which was associated with disease phases and quantitative levels of BCR–ABL1 fusion gene [44, 54, 106]. In addition, TKI treatment in patients led to reduction of immune suppressive cells and exhausted T cell and increase of tumor responding effector T cells [43, 44, 107, 108]. Importantly, restored immune effector cells were critical for deep molecular response, eradication of minimal residual disease and achievement of TFR [43, 44, 45, 46, 106, 109]. We want to point out that in the retroviral‐induced murine CML model in a H8 transgenic mouse background, PD‐L1 expression was detected on CML cells at either the chronic phase or blast crisis by flow cytometry [54]. However, comprehensive transcriptomic analysis of hematopoietic cells along the CML transformation process showed that there was no activation of PD‐L1 expression on CML cells. The inconsistency may be due to different sensitivity of detection methods, differences between transgenic model and retrovirus‐based model, or wild‐type mice and H8 transgenic mice, in which all CML cells expressed the immunodominant GP33 epitope of the lymphocytic choriomeningitis virus glycoprotein on MHC Class I molecules [54, 110]. Despite the different mechanisms proposed underlying immune suppression, both studies have consistently proven the concept of restoring antitumor immunity by targeting T cell exhaustion in CML models, which can be a treatment option to further improve CML patient survival and even achieve TFR.

## 4 Conclusions

This study provides the first comprehensive profiling of the dynamic changes of TME during primary CML induction and secondary transplantation, both of which confirmed an active immune evasion process during CML initiation and progression. Mechanistically, the BCR–ABL1 oncogene orchestrates a global transcriptional reprogramming toward neutrophil differentiation and activation of immune suppression genes, including TGFB1 and ARG2, which enable the neutrophil‐like CML cells efficiently suppressing antitumor tumor immune response mediated by T cells. Further investigation revealed that BCR–ABL1 oncoprotein drives this immune evasion process through TF C/EBPβ, which binds directly to the promoters of TGFB1 and ARG2 in both human and mouse CML models. In consistency, pharmaceutical or genetic inhibition of BCR–ABL1, or its downstream C/EBPβ, TGF‐β and arginase can rescue T cell suppression by neutrophil‐like CML cells. Based on our discovery, we evaluated a combination treatment of targeted therapy using TKI against BCR–ABL1 and immune therapy targeting the PD‐L1/PD‐1 pathway, which led to not only rapid remission, but also delayed relapses in animal model, providing rationality in combining TKI and immune checkpoint inhibitors for TFR of CML.

## 5 Material and Methods

### 5.1 CML Mouse Model and Mouse Engraftment

The primary *SCL:tTA*/*BCR–ABL1* transgenic mice were established by Dr. Daniel G. Tenen and obtained from Dr. Ravi Bhatia [47]. Primary CML was induced by withdrawal of TET from the drinking water of the transgenic mice. For the serial transplantation model, the leukemia cells were retrieved from primary leukemic mice and 5 × 10^6^ BCR–ABL1+ primary cells were engrafted into 8–12‐week‐old female C57BL6 mice with or without irradiation at 6 Gy. All animal experiments were performed under an approved protocol from the Augusta University Institutional Animal Care and Use Committee.

### 5.2 Flow Cytometry Analysis

PB and BM were collected for flow cytometry analysis as described previously [111]. Flow antibodies used in this study are listed in Table S1. All antibodies for flow cytometry were diluted at 1:200. The immune monitoring of primary leukemia induction and subsequent CML cell transplantation has performed at least three times and representative data from one cohort of five mice are presented.

### 5.3 Blood Smear and May‐Grunwald Giemsa Staining

PB smears and Giemsa staining were performed using May‐Grünwald (Sigma; #63590) and Giemsa stain solutions (Sigma; #48900). Images were obtained using an Olympus BX43 Version microscope at a 20× magnification.

### 5.4 Quantitative Real‐Time PCR

The analysis was performed as previously described [111]. The primers sequences used are provided in Table S2. Total RNA was extracted using Trizol reagent from Ly6G+ cells of naïve and BCR–ABL1 BM. cDNA was generated using the High‐Capacity cDNA Reverse Transcription Kit (Applied Biosystems; #4368813). Amplifications were carried out using iTaq Universal SYBR Green Supermix and CFX Opus 96 Real‐Time PCR System (Bio‐Rad). The sequences of primers used are described in Table S2. For TKI treatment of neutrophil‐like CML cells in vitro, the purified Ly6G+ cells were treated overnight with 30 nM ponatinib and 150 nM nilotinib and cells were harvested for RNA isolation and qRT‐PCR analysis. All experiments have been repeated at least three times.

### 5.5 shRNA Knockdown of Target Genes

The pLKO.1 lentiviral vectors containing a shRNA against human *CEBPB*, with shRNA1 (clone ID TRCN0000007440) targeting the 3′UTR, or shRNA2 (TRCN0000007441) targeting the 5′UTR and ORF, and mouse *Cebpb*, with shRNA1 (Clone ID TRCN0000009508) targeting the ORF and 5′UTR, or shRNA2 (Clone ID TRCN0000009509) targeting the ORF, were obtained from Open Biosystems (Huntsville, AL, USA). The scrambled shRNA, Plasmid #1864 was obtained from Addgene (Watertown, MA) with the target sequence CCTAAGGTTAAGTCGCCCTCG. Lentivirus packaging, cell transduction and puromycin (Sigma) selection were performed as previously described [112, 113]. The recovered cells were harvested for RT‐qPCR following standard protocol described above.

### 5.6 ChIP‐qPCR

ChIP‐qPCR was performed using the ChIP Assay Kit following the manufacturer's protocol (Cat#17‐295; Millipore Sigma). Briefly, genomic DNA was shared by sonication to generate fragments of the required length (∼200–1000 bp) after cross‐linked using formaldehyde, then sheared were subjected to immunoprecipitation with antibodies specific for CEBPB (Cat#23431‐1‐AP; Proteintech) or IgG (Cat#30000‐0‐AP; Proteintech). The immune complexes were digested by proteinase K and then purified using Qiaquick PCR purification kits (Cat#28106; Qiagen). qPCR was performed on chromatin using primers spanning the core CEBPB binding sites, and the sequences were provided in Table S2.

### 5.7 IF Staining and Intracellular Staining

Details for IF staining were described in our previous publication [113]. Briefly, magnetically isolated Ly6G+ neutrophils were attached to polysine microscope slides (Thermofisher; #P4981‐001) using a Cytospin Cytocentrifuge. Cells were fixed in 4% PFA, permeabilized with 0.3% Triton X, and blocked with 2% BSA. The cells were then incubated at 4°C overnight with BCR–ABL Monoclonal Antibody (7C6) (1:100; Thermofisher; # MA1‐153) and Ly6G (1:200; Proteintech; #65140‐1‐Ig), followed by incubation with Cross‐Adsorbed Secondary Antibody, Alexa Fluor 488 Goat anti‐Mouse IgG (Invitrogen; #A11001) and Alexa Fluor 546 (Invitrogen; #A11081) Goat anti‐Rat IgG Antibody. Nuclei were stained with DAPI. Cells were flat mounted and examined by confocal microscopy (Leica DMi8). The images were captured at 63× magnification.

### 5.8 T‐Cell Suppression Assay by Neutrophil‐Like CML Cells

Neutrophils were isolated from the BM of naïve mice and the PB or BM of leukemic mice and T cell suppression assay was performed as previously described [60, 111]. Neutrophils were isolated using the Ly6G–biotin (Biolegend; #127604), Anti‐Biotin MicroBeads (Miltenyi Biotec; #130‐097‐046), and LS Separation columns (Miltenyi Biotec; #130‐042‐401). CFSE‐labeled CD4 T cells were cocultured with neutrophils for 4 days in the presence of anti‐CD3/anti‐CD28 Dynabeads (Gibco; #11452D). T cell proliferation was then analyzed using flow cytometry for CFSE by gating on the CD4 population. For blockage of BCR–ABL1, TGF‐β, or arginase inhibition, 30 nM ponatinib (AP), 150 nM nilotinib, 10 µM galunisertib, or 30 µM Nor.NOHA (Sigma–Aldrich) was used in the pretreatment of neutrophils for 8 h, respectively. After washing, the cells were added to the T cell coculture.

### 5.9 Animals and Treatment Schedule

All animal experiments were performed under an approved protocol from the Augusta University Institutional Animal Care and Use Committee. 8–10‐week‐old female mice were irradiated and then injected with primary BCR–ABL1 expressing cells at 5 × 10^6^ per mouse via the tail vein. For in vivo treatment, 40 days after transplantation of primary leukemia cells, mice with successful leukemia engraftment were randomized to 5 mice/group for treatment with vehicle, ponatinib alone (6 mg/kg/day; MCE; #HY‐12047) and ponatinib combined with an anti‐PD1 antibody (100 µg/mouse; Biolegend; #135248). All mice were included since 100% of the transplanted mice developed leukemia. All treatments were performed every other day for 4 weeks by intraperitoneal (IP) injection. Immune monitoring was performed as described above. The mice were sacrificed after 5 weeks of treatment discontinuation.

### 5.10 RNA‐Seq and Analysis

RNA‐Seq data for transgenic mice undergoing leukemia induction and different treatment were obtained from GEO database accession number GSE244990 [55], which includes bulk RNA‐Seq gene expression profiling of time‐sequential PB samples from cohorts of Off TET BCR–ABL‐inducible transgenic mice. We include data from three experimental cohorts of mice that were sampled weekly for 18 weeks or until mice became moribund with disease: On TET control naïve mice without BCR–ABL expression; Off TET CML mice with BCR–ABL induction and CML progression; and TKI treatment cohorts where TKI nilotinib was administrated in leukemia bearing mice for a 4‐week treatment window during Week 6 to Week 9.

### 5.11 Statistical Analysis

The Student's *t*‐test was performed for comparison between two groups, whereas ANOVA was used for comparison of three or more groups. ns represents not significant; *p < 0.05, **p < 0.01, ***p < 0.001, ****p < 0.0001.

## Author Contributions

TH and BC conceived and planned the experiments. Experiments were performed by BC, XL, YL, HF, CL, XF, AM, SM, TZ, GW, GZ, MY, and TH. Bioinformatic analysis was performed by HD and TH. JC helped with data interpretation and manuscript revision. TH wrote and revised the manuscript. All authors have read and approved the final manuscript.

## Funding

This work was supported by grant CA076167 from the National Institutes of Health. This research was also supported in part by the Paceline Award and the Shared Resources Assistance Program (ShRAP) from the Georgia Cancer Center.

## Ethics Statement

No patients are involved with the current research. The mouse experiments performed were approved by the Augusta University Institutional Animal Care and Use Committee under the protocol #2008‐0153.

## Conflicts of Interest

All the authors declare no conflicts of interest.

## Supporting information




**Supporting File 1**: mco270747‐sup‐0001‐SuppMat.docx

## Data Availability

All data that support the findings of this study are included within this paper and its Supplementary Information files. The RNA‐Seq data are publicly available in GEO databases.
