# Supplementary material for: BCR–ABL1 Drives Transcriptional Reprogramming of Chronic Myeloid Leukemia Cells for Immune Evasion Through C/EBPβ
Source: MedComm (2020). 2026 Apr 26;7(5):e70747. doi: 10.1002/mco2.70747 (PMC13111919; doi:10.1002/mco2.70747)
Supplement: Supplementary file 1 — Supporting File 1: mco270747‐sup‐0001‐SuppMat.docx [file MCO2-7-e70747-s001.docx]

**BCR-ABL1 drives transcriptional reprogramming of Chronic Myeloid Leukemia cells for immune evasion through C/EBPβ**

**Running title: BCR-ABL1 drives immune evasion through C/EBPβ**

Xiaocui Lu^1, 2, #^, Hui Fang^1, 2, #^, Yuan Liu^1,^ ^#^, Chang Liu^1, 2^, Xuexiu Fang^2^, Atsuko Matsunaga^2^, Stephanie F. Mori^2^, Ting Zhang^2, 3^, Gavin Wang^2^, George I. Zhou^2^, Miao Yu^2^, Haocheng Ding^4^, Jorge Cortes ^2^, Bo Cheng^1, *^ and Tianxiang Hu^2, 5, *^

^#^These authors contributed equally to the project.

*Co-corresponding authors.

Corresponding authors: Tianxiang Hu, [tihu@augusta.edu](mailto:tihu@augusta.edu)

Bo Cheng, [chengbo@znhospital.cn](mailto:chengbo@znhospital.cn)

^1.^ Department of Stomatology, Zhongnan Hospital of Wuhan University, Wuhan, China 430071

^2.^ Georgia Cancer Center, 1410 Laney Walker Blvd, Augusta, GA 30912.

^3.^ Department of Dermatology, Tianjin Academy of Traditional Chinese Medicine Affiliated Hospital, Tianjin, China 300193

^4.^ Department of Biostatistics, Data Science and Epidemiology, School of Public Health, Augusta University, 30912 Augusta, GA, USA

^5.^ Department of Biochemistry and Molecular Biology, Medical College of Georgia, Augusta, GA 30912


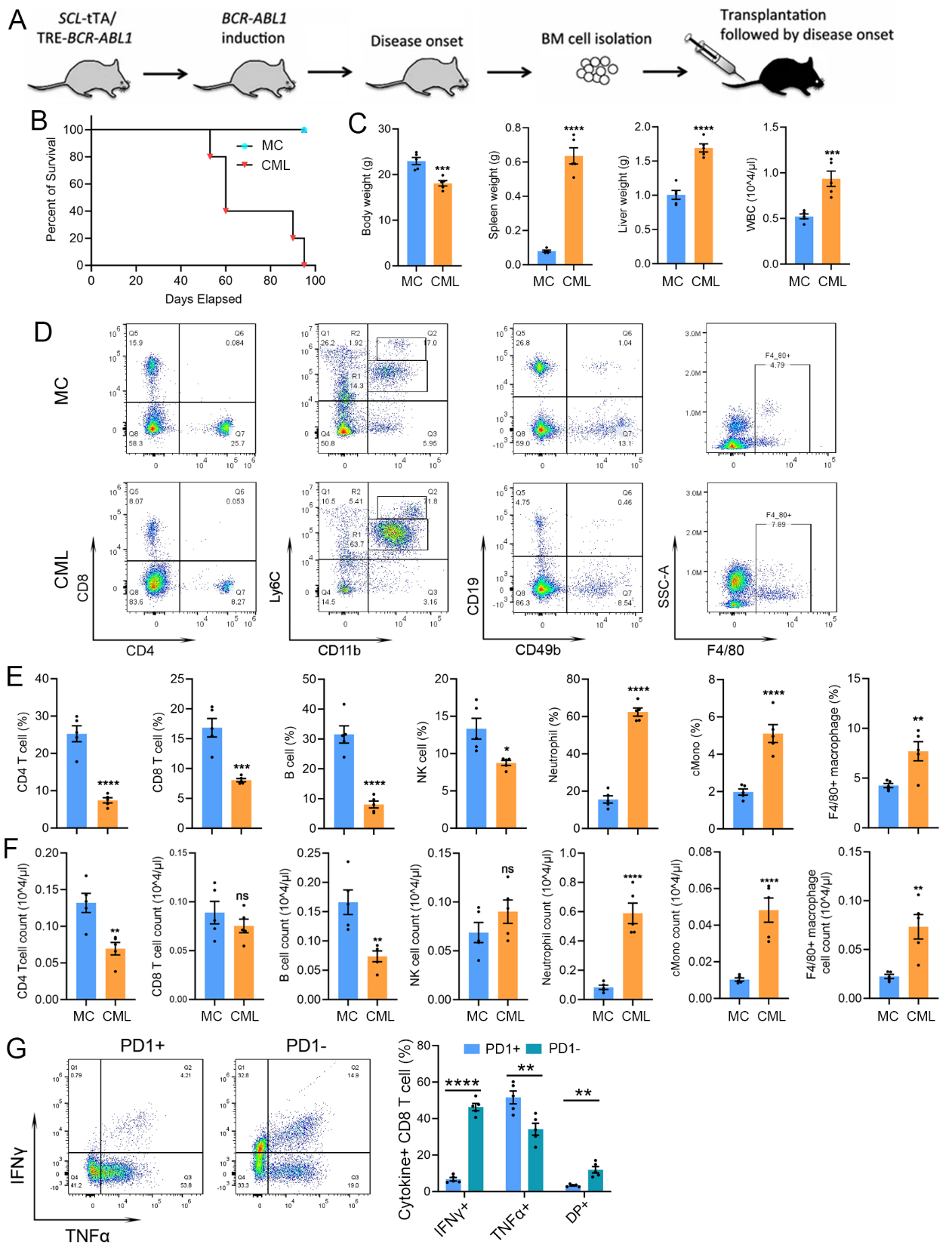


**Figure S1.** Schematic diagram summarizing the primary CML induction in transgenic mice and subsequential CML cell transplantation model (A). The mimic control (MC) group went through 6 Gy irradiation but without leukemia cell transplantation. Survival of the recipient mice (n=5) with and without transplantation of the primary CML cells (B). The body, spleen and liver weights and WBC of mice from different mouse cohorts at time of sacrifice (C). Representative dot plots for flow cytometry analysis of peripheral blood samples (D) and corresponding statistical data for cell percentages (E) and cell counts (F) of indicated immune cells. Flow cytometry measurement of production of IFNγ and TNFα by the PD1+ and PD1- CD8 T cells from leukemia mice (G). The data presented are representative results from at least three repeated experiments. The student's t test was performed for comparison between two groups. ns represents not significant; * p < 0.05, ** p< 0.01, *** p < 0.001, **** p < 0.0001.


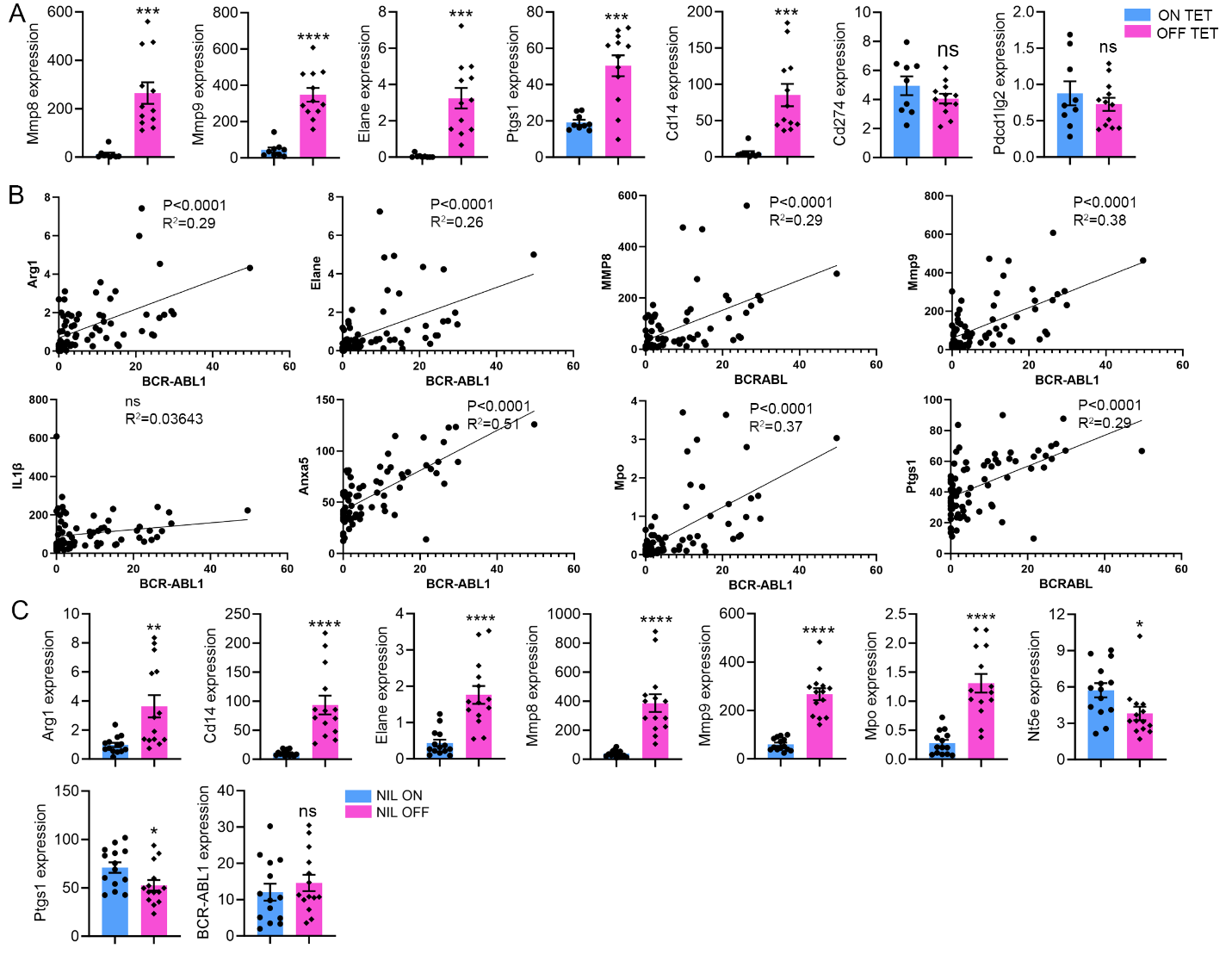


**Figure S2.** (A) The expression levels of the selected genes in Fragments Per Kilobase of transcript per Million mapped reads (FPKM) in samples from naïve control (ON TET) and leukemic mice (OFF TET). (B) The correlations between the mRNA expression levels of BCR-ABL1 and selected immune suppression genes were analyzed using the gene expression profiling data of transgenic mice along CML progression. (C) The expression levels of the selected genes in mouse samples collected from the same mice under the Nilotinib treatment (NIL ON) and after discontinuation (NIL OFF).

**
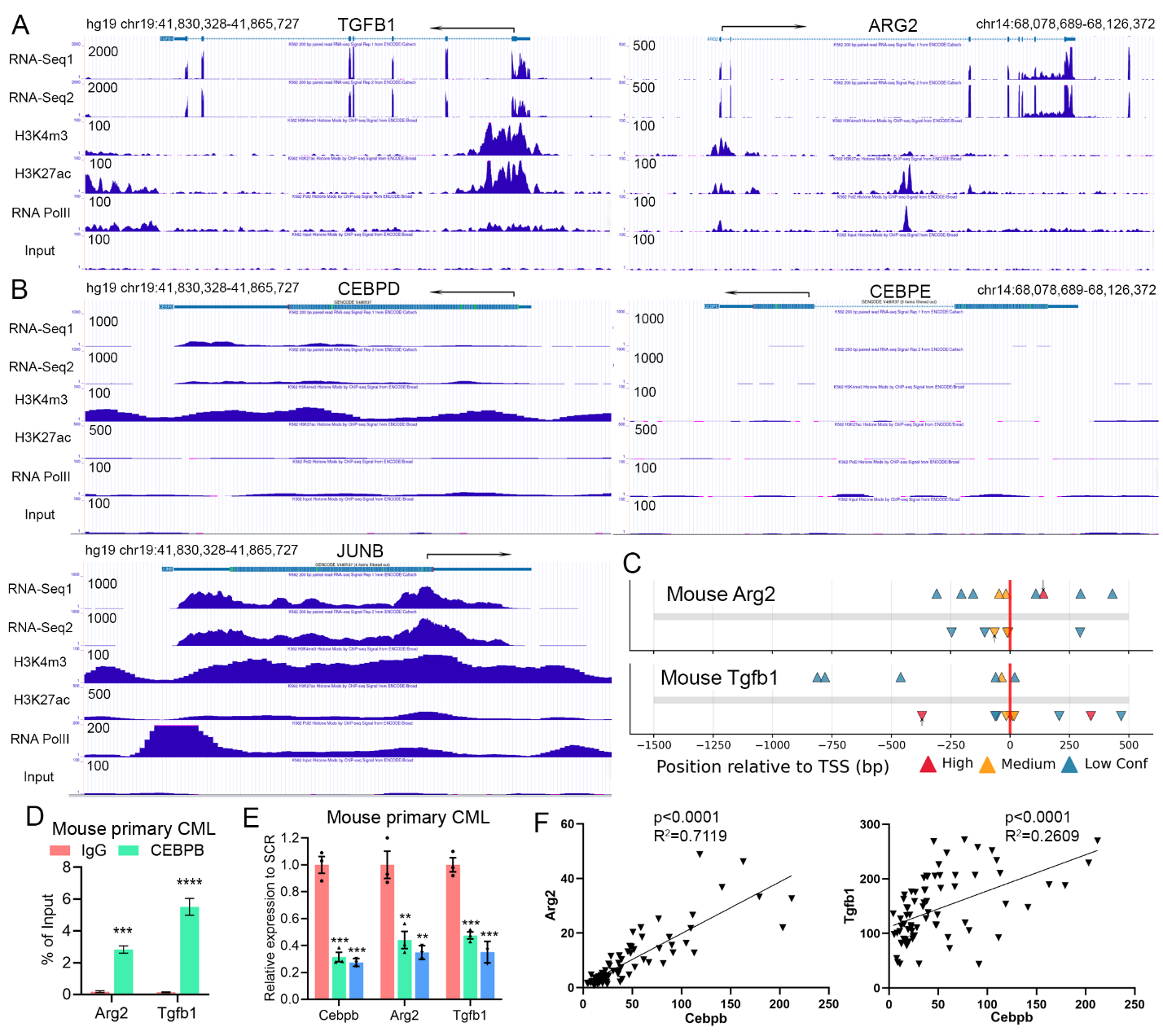
 Figure S3.** Genome browser snapshot of the indicated gene loci showing tracks of RNA-Seq data, and ChIP-Seq data with histone markers H3K4m3 and H3K27ac, and RNA PolII from K562 cells. (A) for immune suppression genes and (B) for transcription factors in neutrophils. Diagram showing the locations of the predicted Cebpb sites in the promoter regions of mouse Arg2 and Tgfb1 genes (C). ChIP-qPCR quantification of the enrichment of target genomic DNA relative to the total input DNA after pulling down using CEBPB antibody or IgG control (D). qRT-PCR detection of mouse gene expression in BCR-ABL1 transformed primary CML cells transduced with lentivirus carrying shRNAs targeting Cebpb or the SCR control (E). Correlations between the mRNA expression levels of Cebpb and Arg2 or Tgfb1 (F). TSS, Transcription starting site. Red, yellow and blue triangles represent those predicted binding motifs with high, medium and low confidence, respectively.

| **Table S1. Antibodies for flow cytometry analysis.** | | |
| --- | --- | --- |
| **Antibody** | **Supplier** | **Catalog Number** |
| CD4-APC | Biolegend | #100412 |
| CD8α-PE/Cy7 | Biolegend | #100722 |
| Ly6C-PE | Biolegend | #128008 |
| CD11b-PerCP/Cy5.5 | Biolegend | #101228 |
| Ly6G-APC/Cy7 | Biolegend | #127624 |
| CD19-PE/Cy7 | Biolegend | #152418 |
| CD49b-APC | Biolegend | #103516 |
| F4/80-PE | Biolegend | #123110 |
| PD-L1-PE/Cy7 | Biolegend | #124314 |
| CD8a-FITC | Biolegend | #100706 |
| CD4-PE/Cy7 | Biolegend | #100421 |
| PD1-PE | Biolegend | #135205 |
| Ly6C-APC | Biolegend | #128016 |
| F4/80-APC/Cy7 | Biolegend | #123118 |
| CD19-BV421 | Biolegend | #115538 |

| **Table S2. Primer sequences used for real-time quantitative PCR (qRT-PCR) and ChIP-qPCR analysis** | |
| --- | --- |
| **(A) Primer pairs for qRT-PCR** | |
| **Name** | **Sequence** |
| hBCRABL1qFP1 | CAGCATTCCGCTGACCATCA |
| hBCRABL1qRP1 | AACGAGCGGCTTCACTCAG |
| hARG2qFP1 | ACCTGATAGTGAATCCACGCT |
| hARG2qRP1 | CATGGGCATCAACCCAGAC |
| hTGFB1qFP1 | GGCCAGATCCTGTCCAAGC |
| hTGFB1qRP1 | GTGGGTTTCCACCATTAGCAC |
| hSTAT5AqFP1 | CCAGTACCAGGAGAGCCTGA |
| hSTAT5AqRP1 | AGACACCTGCTTCTGCTGGA |
| hSTAT5BqFP1 | GATCAAGCTGGGGCACTATG |
| hSTAT5BqRP1 | CTCGGACCAACCTCTGTTCA |
| mBCRqFP1 | AATGAGCATCCCTGTGTCGG |
| mBCRqRP1 | CTGGGGCCGTCATTCTCTTT |
| mcAblqFP1 | CCTCCTTTGCTGAAATCCACC |
| mcAblqRP1 | ACTGTCCAGTGCATCGCTTTC |
| mArg1qFP1 | TGTCCCTAATGACAGCTCCTT |
| mArg1qRP1 | GCATCCACCCAAATGACACAT |
| mArg2qFP1 | TCCTCCACGGGCAAATTCC |
| mArg2qRP1 | GCTGGACCATATTCCACTCCTA |
| mTgfb1qFP1 | TGGAAATCAACGGGATCAGC |
| mTgfb1qRP1 | AGTTGGTATCCAGGGCTCTC |
| mActbFP1 | TGGCTCCTAGCACCATGAA |
| mActbRP1 | CTCAGTAACAGTCCGCCTAGAAGCA |
|  |  |
| **(B) Primer pairs for ChIP-qPCR** | |
| **Name** | **Sequence** |
| hARG2promFP1 | TTACTGGCCAGACCCGGATA |
| hARG2promRP1 | TTAGGGACATGATCCGCAGC |
| hTGFB1promFP1 | GGCTTAATCCGGGGGATGAG |
| hTGFB1promRP1 | CCCCATGTTGACAGACCCTC |
| mArg2promFP1 | GGAGAGCTGTAGTCCTCCGA |
| mArg2promRP1 | GAATTTGCCCGTGGAGGAGA |
| mTgfb1promFP1 | TCTCAGACGTCGGTCTCCTT |
| mTgfb1promRP1 | TCTTCATCTTAGCGTGGGCG |
